# 

**Published:** 1885-09-20

**Authors:** 


### TABLE OF CONTENTS.

ORIGINAL AND SELECTED ARTICLES:

Diagnosis of Incipient Phthisis, by E. van Goidtsnoven, M.D., Atlanta, Ga. Read before the Atlanta Society of Medicine, August. 1885, 321. A case of Bright's Disease.—Recovery, by P. R. Cortelyou, of Georgia, 328. A Case of Retention of Urine Caused by a Stone in the Bladder, by G. E Flowers, M.D., of Ga.; On Repeated Doses of Castor Oil, Especially in Certain Skin Diseases in Children, by L. Duncan Bulcley, A.M., M.D., of New York, 330. On the Anæsthetic Action of Apomorphine, 336.

ABSTRACTS AND GLEANINGS:

The Communicability of Scarlet Fever, 337. Carbuncle Treated with Oleate of Morphia, 338. Chloroform Water, 339. How to Take a Pill, 340. A New Treatment for Goitre, 341. Cholera Inoculation, 342. Prostatotomy, 343 The Abortive Treatment of Typhoid Fever with Naphthalin; Reduced Iron in the Treatment of Anæmia, 344. Painless Removal of Toe nail; Atonic Dyspepsia, 345. Tamponing the Nasal Fossæ with Plugs Soaked in Turpentine; Hygrine or Cocaine, 346. Sulphate of Magnesia in Dropsy; Corrosive Sublimate in Veneral Warts; Cold in the Head, 347. Piles and Fissured Anus; Morphia in Cholera Infantum; Tannic Acid Injections in Cholera, 348. Chancre; Treatment of Intermittent Fever by the Intermittent Current; Irritable Bladder in Old Men; Salt Baths in the Treatment of Fever; Buzzing Noise from Quinine, 349. Piles, thei Hypodermic Injection; Peculiar Results of Bee-Stings; Paralysis Agitans; Sugar in Phthisis and Dysentery, 350.

SCIENTIFIC ITEMS:

A New Swedish Invention; Climatology of the Puget Sound Country; A New Use for Toads, 351. Animal Eccentricities; A Surgical Instrument; A Life Saving Apparatus; A Slicer; A Machine, 352.

PRACTICAL NOTES AND FORMULÆ.

For Vomiting, Cholera, etc.; Earache, 353. Application for Inflamed Surfaces; Salicylate Potassium in Acute Rheumatism; For Burns; Stimulating Anodyne Liniment, 354. Nocturnal Emissions; Antidote for Rhus Poisoning, 355.

EDITORIALS AND MISCELLANEOUS.

Notice; Dr Searcy Dead; The Inoculation for Cholera; American Rhinological Association: Ethics and Jurisprudence; New Orleans Exposition, 356. The Southern Medical College; The Eclectic Journals; Number of Lawyers, Ministers and Doctors in the United States; Books and Pamphlets Received, 357. Receipted; Special Notices, 360.

